# Neuroimaging spectrum of myelin oligodendrocyte glycoprotein antibody-associated disease with brain involvement: description of various cerebral syndromes

**DOI:** 10.1007/s00247-025-06342-y

**Published:** 2025-08-11

**Authors:** Young Hun Choi, Woo Joong Kim, Byung Chan Lim, Il Han Yoo, Yeon Jin Cho, Seunghyun Lee, Jae-Yeon Hwang, Jung-Eun Cheon

**Affiliations:** 1 Department of Radiology, Seoul National University College of Medicine, Seoul, Republic of Korea; 2 Innovative Medical Technology Research Institute, Seoul National University Hospital, Seoul, Republic of Korea; 3 Department of Pediatrics, Seoul National University Children’s Hospital, Seoul, 03080 Republic of Korea; 4 Department of Pediatrics, Seoul National University College of Medicine, Seoul, Republic of Korea; 5 Department of Pediatrics, College of Medicine, The Catholic University of Korea, St. Vincent’s Hospital, Suwon, Republic of Korea; 6 Department of Radiology, Seoul National University Children’s Hospital, Seoul, Republic of Korea; 7 Department of Radiology, Seoul National University College of Medicine, Seoul, Republic of Korea; 8 Institute of Radiation Medicine, Seoul National University Medical Research Center, Seoul, Republic of Korea

**Keywords:** Acute disseminated encephalomyelitis, Child, Demyelinating diseases, Encephalitis, Magnetic resonance imaging, Myelin oligodendrocyte glycoprotein

## Abstract

**Background:**

Myelin oligodendrocyte glycoprotein antibody-associated disease (MOGAD) is a notable cause of acquired central nervous system inflammatory disorders in children.

**Objective:**

This study aimed to characterize the neuroimaging spectrum of pediatric MOGAD with brain involvement.

**Materials and methods:**

In this retrospective, single-center study, 55 children diagnosed with MOGAD involving the brain between January 2010 and October 2020 were included. Clinical data and neuroimaging—brain and spinal magnetic resonance imaging (MRI) at presentation—were reviewed. Imaging patterns were categorized into six radiologic phenotypes: acute disseminated encephalomyelitis (ADEM), cerebral cortical encephalitis, aseptic meningitis, tumefactive demyelinating lesion, cerebellitis/brainstem encephalitis, and miscellaneous. Imaging features were further analyzed in the ADEM subgroup.

**Results:**

ADEM was the most common phenotype (39 of 55 patients, 71%), though atypical features were frequent, with 62% showing at least one atypical MRI finding. Unlike classic ADEM with large confluent white matter lesions, MOGAD-associated ADEM often showed small (31%) or subcortical (44%) white matter lesions. Spinal lesions typically appeared as longitudinally extensive myelitis with central gray matter involvement. Other phenotypes included cortical encephalitis (three patients), aseptic meningitis (six), tumefactive demyelinating lesions (three), cerebellitis/brainstem encephalitis (two), and two miscellaneous patterns. Non-ADEM phenotypes presented at an older age than ADEM (11.5 years vs. 5.2 years, *P* < 0.01), with a threshold of 7.6 years.

**Conclusion:**

Pediatric MOGAD with brain involvement presents a range of imaging patterns. ADEM is most frequent but often displays atypical features. Non-ADEM phenotypes tend to occur in older children.

**Graphical Abstract:**

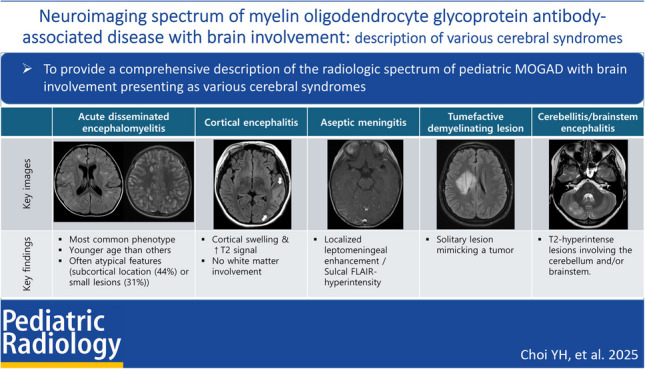

**Supplementary Information:**

The online version contains supplementary material available at 10.1007/s00247-025-06342-y.

## Introduction

Myelin oligodendrocyte glycoprotein (MOG), a surface antigen located on oligodendrocyte membranes and the outermost layer of myelin sheaths, has been recognized over the past decade as a key target in immune-mediated inflammation of the central nervous system (CNS) [1]. MOG antibody-associated disease (MOGAD) has emerged as a leading cause of acquired demyelinating syndromes [2–8], manifesting as acute disseminated encephalomyelitis (ADEM), neuromyelitis optica spectrum disorder (NMOSD) without anti-aquaporin-4 antibodies, optic neuritis, and transverse myelitis in both pediatric and adult populations [4, 5]. The clinical and radiological features of MOGAD are increasingly recognized as distinct from those of multiple sclerosis and aquaporin-4 antibody-positive demyelinating disease [5, 9–12]. Early recognition of MOGAD is essential, as the condition typically responds well to corticosteroid therapy, whereas treatment delays may result in significant long-term neurological impairment.

Recently, the International MOGAD Panel introduced new diagnostic criteria for MOGAD [13]. These criteria emphasize the presence of MOG-IgG (immunoglobulin G) antibodies and magnetic resonance imaging (MRI) findings, including optic neuritis, transverse myelitis, and brain, brainstem, or cerebellar syndromes. According to the guideline, MRI plays a central role in interpreting MOG antibody test results. Furthermore, radiologic suspicion of MOGAD can influence the decision to initiate MOG-IgG testing, especially considering its variable availability and delayed turnaround time. Since universal screening for MOG-IgG is not recommended in all cases of CNS demyelination [13], it is crucial to accurately define and describe the imaging features associated with MOGAD.

Unlike optic neuritis and transverse myelitis, MOGAD involving the brain and brainstem exhibits considerable heterogeneity in both clinical presentation and MRI findings. Brain involvement in MOGAD has traditionally been associated with pediatric ADEM or “ADEM-like” syndromes. However, over the past decade, the recognized radiological spectrum of MOGAD has broadened, with emerging patterns that do not align with the classical definition of ADEM. Even the most recent diagnostic criteria do not fully capture this range of brain imaging presentations, raising concerns about potential underdiagnosis using current guidelines [14]. This issue is particularly relevant in children, as brain involvement is more frequently observed in the pediatric population compared to adults [15], and approximately 5–10% of pediatric MOGAD cases are reported to present with atypical features [16].

Furthermore, while previous studies have occasionally described radiologic findings in MOGAD with brain involvement—such as ADEM, cortical encephalitis, and other manifestations [4, 6, 9, 17, 18]—no prior research has systematically focused on MRI characteristics or conducted a comprehensive evaluation of neuroradiological features specific to pediatric MOGAD with brain involvement. Therefore, the objective of this study was to provide a detailed overview of the radiologic spectrum of pediatric MOGAD presenting with various cerebral syndromes, to enhance understanding of this disease subgroup.

## Materials and methods

### Study population

This retrospective study was approved by the Institutional Review Board of our hospital, and the requirement for informed consent was waived.

Children under 18 years of age diagnosed with anti-MOGAD involving the brain were identified at xxx hospital between January 2010 and October 2020. The diagnosis of MOGAD was confirmed in patients who exhibited core clinical brain syndromes and tested clearly positive for MOG-IgG using a live cell-based assay [13, 19]. From this group, only those with brain, brainstem, and/or cerebellar abnormalities evident on the initial brain MRI were included in the analysis. Ultimately, all selected patients fulfilled the criteria for “definite antibody-positive” autoimmune encephalitis as defined by the Autoimmune Encephalitis International Working Group [20]. According to these criteria, the diagnosis requires that previously healthy children present with an acute or subacute onset (within 3 months) of at least two neurological and/or psychiatric symptoms—such as focal or generalized neurological deficits, cognitive impairment, developmental regression, abnormal movements, psychiatric symptoms, and/or seizures—along with paraclinical evidence of neuroinflammation from MRI, cerebrospinal fluid (CSF) analysis, or brain biopsy, and detectable autoantibodies in the serum and/or CSF. Patients presenting with isolated optic nerve or spinal cord involvement were excluded. Basic demographic and clinical information—including sex, age at onset, initial clinical manifestations, history of seizures, and CSF findings—were obtained through a review of electronic medical records.

### Myelin oligodendrocyte glycoprotein antibody testing

Serum samples were obtained from patients within 1 month of symptom onset and stored in a liquid nitrogen tank following centrifugation. Detection of MOG antibodies was performed qualitatively using a live-cell-based indirect immunofluorescence assay. To assess antibody positivity, the median fluorescence intensity (MFI) ratio—representing the relative fluorescence signal between MOG antibody-expressing cells and healthy controls—was used. Cutoff values for MOG antibody status, based on receiver operating characteristic (ROC) analysis from a previous study, were defined as follows: negative (≤ 2.60), borderline (2.60–3.65), and positive (> 3.65). Only patients with MFI ratios exceeding 3.65 were included in the analysis. The serum MOG-IgG assay followed the method previously described in the same study [19]. Briefly, MOG-expressing human embryonic kidney (HEK) 293 cells were seeded onto eight-well chambered slides (SPL Life Sciences, Pocheon, Korea) and incubated overnight at 37℃ in 5% CO_2_. The next day, cells were blocked using a buffer containing 1 × phosphate-buffered saline (PBS) and 5% bovine serum albumin for 1 h at room temperature. Patient serum, diluted 1:20, was added to each well, and slides were incubated for 2 h at room temperature. Cells were then fixed with 2% paraformaldehyde for 45 min. After washing, cells were stained with Alexa-594 conjugated anti-human IgG (Jackson ImmunoResearch, West Grove, PA; diluted 1:2,000 in 1 × PBS) for 1 h in the dark at room temperature. Following three washes, slides were mounted using VECTASHIELD® antifade reagent with DAPI (Vector Laboratories, Burlingame, CA). All experiments were conducted in duplicate, and membrane-bound green or red fluorescence was visually evaluated by the investigators.

### Evaluation of initial magnetic resonance imaging findings

All brain MRI scans obtained during the initial acute episode were retrospectively reviewed in consensus by two experienced pediatric radiologists (xxx and xxx, with 26 years and 16 years of experience, respectively). The evaluation included standard sequences and all additional available sequences: axial T2-weighted, axial fluid-attenuated inversion recovery (FLAIR), axial and sagittal T1-weighted, diffusion-weighted imaging, gradient-recalled echo or susceptibility-weighted imaging, and post-contrast axial T1-weighted sequences. Spine MRIs, when available, were also reviewed. The basic spine sequences consisted of sagittal and axial T2- and T1-weighted images, along with post-contrast sagittal and axial T1-weighted sequences.

Patients were classified into six subtypes according to the predominant pattern of abnormalities observed on MRI:

1. Acute disseminated encephalomyelitis (ADEM): multifocal, disseminated T2-hyperintense lesions. These typically appear as large, confluent, bilateral but asymmetric cerebral white matter abnormalities.
2. Cerebral cortical encephalitis: T2 hyperintensity and gyral swelling, with or without adjacent leptomeningeal enhancement. Initial presentation lacks white matter involvement and therefore does not meet ADEM classification criteria [7].
3. Aseptic meningitis: localized leptomeningeal enhancement or sulcal FLAIR hyperintensity without associated brain parenchymal changes.
4. Tumefactive demyelinating lesion: a solitary lesion larger than 2 cm, which may radiologically mimic a neoplasm.
5. Cerebellitis/brainstem encephalitis: T2-hyperintense lesions predominantly or exclusively involving the cerebellum and/or brainstem.
6. Miscellaneous: minor imaging patterns not fitting into the above subtypes:
  - Leukodystrophy-like phenotype: large, nearly symmetric confluent white matter lesions resembling leukodystrophy [21]
  - Multiple sclerosis-like phenotype: well-circumscribed periventricular lesions aligned with the long axis of the corpus callosum [22].

### Magnetic resonance imaging analysis in the acute disseminated encephalomyelitis subgroup

In patients classified under the ADEM phenotype, lesions were categorized by location in the following regions: supratentorial white matter (including subcortical white matter, deep white matter, and the corpus callosum), deep gray matter (such as the thalamus and basal ganglia), brainstem, cerebellum, and spinal cord. A widespread score was assigned based on the number of these five anatomical regions involved, with scores ranging from 1 to 5. A higher score indicated a greater distribution of lesions.

**Fig. 1:**
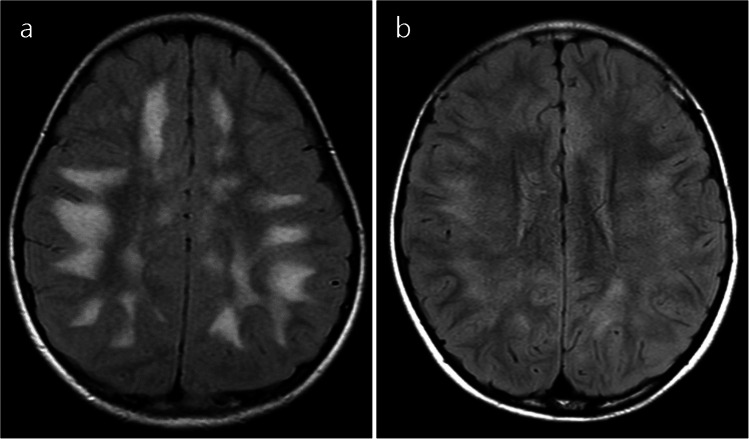
Typical acute disseminated encephalomyelitis (ADEM). Typical ADEM was characterized by bilateral, diffuse, poorly demarcated, large (> 1–2 cm) confluent lesions primarily affecting the cerebral white matter. **a** Axial fluid-attenuated inversion recovery image from a 5-year-old girl with myelin oligodendrocyte glycoprotein antibody-associated acute disseminated encephalomyelitis (MOG-ADEM) demonstrates bilateral, diffuse, large confluent white matter lesions. **b** Axial fluid-attenuated inversion recovery image from a 6-year-old boy with MOG-ADEM shows a similar pattern of bilateral, diffuse, confluent white matter lesions, although the margins are less distinct compared to those in (**a**)

**Fig. 2:**
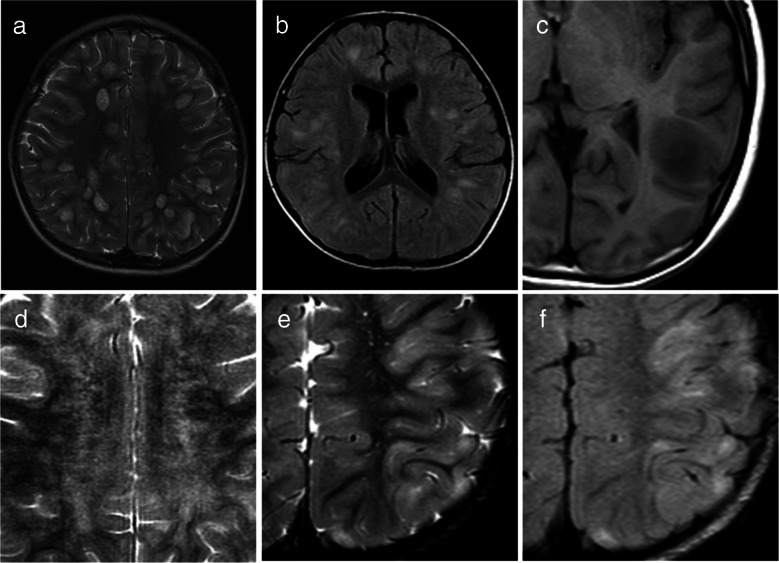
Representative magnetic resonance imaging features considered atypical for acute disseminated encephalomyelitis. **a** Axial T2-weighted image in a 5-year-old boy with myelin oligodendrocyte glycoprotein antibody-associated acute disseminated encephalomyelitis (MOG-ADEM) shows well-demarcated lesions predominantly involving subcortical white matter. **b** Axial fluid-attenuated inversion recovery image in a 5-year-old girl with MOG-ADEM shows small (< 2 cm) lesions. **c** Axial T1-weighted image in an 11-year-old girl with MOG-ADEM shows a T1-hypointense lesion (*arrow*) compared to gray matter. **d** Axial T2-weighted image in a 2-year-old girl with MOG-ADEM shows white matter lesions oriented perpendicular to the ventricles (*arrows*), appearing as laterally extending striations. **e**, **f** Axial T2-weighted (**e**) and fluid-attenuated inversion recovery (**f**) images in a 3-year-old boy with MOG-ADEM show cortical lesions (*arrows*)

Typical ADEM on brain MRI was defined by the presence of bilateral, diffuse, poorly demarcated, large (> 1–2 cm) confluent lesions primarily affecting the cerebral white matter (Fig. 1) [23]. The following imaging features were considered atypical for ADEM [24]: unilateral lesions, well-defined lesions, exclusively small lesions (all < 2 cm), T1-hypointense lesions, lesions oriented perpendicular to the ventricles, lesions predominantly affecting the subcortical white matter more than the periventricular or deep white matter, and definite cortical involvement (Fig. 2). T1-hypointense lesions were defined as those appearing hypointense compared to gray matter on T1-weighted imaging. When evaluating lesion size, confluent lesions were regarded as large even if composed of multiple smaller aggregating lesions. Subcortical white matter lesions with suspected involvement of the adjacent cortex were not classified as cortical lesions.

Spinal cord involvement in ADEM cases was also assessed, including evaluation of the longitudinal extent of the lesion, axial imaging characteristics, and whether the conus medullaris was affected.

### Statistical analysis

Discrete variables in the study population were expressed as medians with ranges, while categorical variables were reported as counts and percentages. Specific MRI findings were described qualitatively. To compare the imaging characteristics of MOG-associated ADEM in this study, imaging features were evaluated against those reported by Baumann M et al. [24] using Fisher’s exact test. The age distribution between the ADEM group and the non-ADEM group (i.e., all phenotypes excluding ADEM) was assessed using the Mann–Whitney *U* test. Additionally, the odds ratio was calculated through logistic regression analysis. ROC curve analysis and Youden’s *J* statistics were employed to identify the optimal age threshold for distinguishing ADEM from non-ADEM phenotypes. A *P*-value of < 0.05 was considered statistically significant. All statistical analyses were performed using MedCalc software (MedCalc, Mariakerke, Belgium).

## Results

A total of 55 patients were included in the study (25 boys and 30 girls; median age, 6 years; range, 15 months to 17 years) (Table 1). Initial clinical symptoms included seizures (*n* = 20), gait instability or ataxia (*n* = 34), psychiatric manifestations (*n* = 15), dysphasia (*n* = 19), movement disorders (*n* = 18), brainstem dysfunction (*n* = 8), focal weakness (*n* = 29), memory dysfunction (*n* = 2), and altered consciousness (*n* = 24). Fever exceeding 38 °C was present in 36 patients (65.5%), and headache was reported in 16 (29.0%). A detailed breakdown of symptoms by MRI subtype is provided in the supplementary table. The median interval between symptom onset and initial MRI was 2 days. CSF profiles at presentation were available for 52 patients. The median CSF protein concentration was 41.5 mg/dL (range, 19–122), with elevated levels noted in 26 patients (50%). The median CSF white blood cell count was 20 cells/mm^3^ (range, 0–556), with elevated counts in 41 patients (78.9%). Aquaporin-4 antibody testing was performed in 36 patients, all of whom were seronegative. N-Methyl-d-aspartate receptor antibody testing was conducted in 15 patients, with all results negative.

**Table 1:** Summary of clinical demographic data of the patients

	% (proportion) or median (minimum–maximum)
Demographic profile	
Age of onset (years)	5.85 (1.32–17.79)
Female sex (%)	54.5 (30/55)
Symptom profile	
Seizure (%)	36.4 (20/55)
Gait instability/ataxia (%)	61.8 (34/55)
Psychiatric symptoms (%)	27.3 (15/55)
Dysphasia (%)	34.6 (19/55)
Movement disorder (%)	32.7 (18/55)
Brainstem dysfunction (%)	14.5 (8/55)
Focal weakness (%)	52.7 (29/55)
Memory dysfunction (%)	3.6 (2/55)
Altered consciousness (%)	45.4 (24/55)
Associated symptoms	
Fever (%)	65.5 (36/55)
Headache (%)	32.7 (18/55)
Laboratory findings	
CSF pleocytosis^a^ (%)	78.9 (41/52)
High pleocytosis^a^ (%)	23.1 (12/52)
CSF protein elevation^b^ (%)	50.0 (26/52)
AQP4 Ab	0 (0/36)
NMDA-R Ab	0 (0/15)
CSF oligoclonal band^c^	26.7 (8/30)
Imaging	
Onset to MRI (days)	2.0 (0–13)

*AQP4* Ab anti-aquaporin-4 antibody, *CSF* cerebrospinal fluid, *NMDA-R Ab* N-methyl-d-aspartate receptor antibody, *MRI* magnetic resonance imaging

^a^Pleocytosis, CSF WBC > 5 cells/μL; high pleocytosis, CSF WBC > 100 cells/μL

^b^CSF protein > 40 mg/dL

^c^CSF oligoclonal band ≥ 2

Radiologic phenotypes in the MOGAD cohort were classified as follows: ADEM in 39 patients, cortical encephalitis in three, aseptic meningitis in six, tumefactive demyelinating lesions in three, cerebellitis/brainstem encephalitis in two, other patterns in two patients (one leukodystrophy-like and one multiple sclerosis-like).

**Fig. 3:**
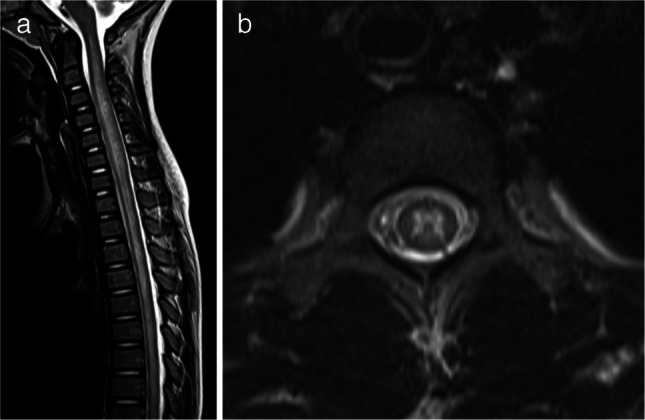
Magnetic resonance images illustrate myelitis in myelin oligodendrocyte glycoprotein antibody-associated acute disseminated encephalomyelitis in a 6-year-old girl. **a** Sagittal T2-weighted image shows a longitudinally extensive spinal cord lesion. **b** Axial T2-weighted image at the upper thoracic level reveals a hyperintense lesion involving the central gray matter (H-sign)

For patients with the ADEM phenotype, the mean widespread score was 2.7 (median 2, range 1–5). In seven cases, only multifocal supratentorial white matter lesions were observed, corresponding to a widespread score of 1. Spinal MRI was performed in 22 of the 39 patients (56%), and myelitis was identified in 10 of these 22 patients (45%). Except for one patient, nine cases showed longitudinally extensive lesions involving the central gray matter on axial images (Fig. 3). Conus involvement was identified in two out of the nine patients; in one patient, evaluation of the conus was not possible due to incomplete MRI coverage. A summary of the MRI distribution of affected regions in MOGAD patients with the ADEM phenotype is shown in Table 2. Typical ADEM patterns (Fig. 1) were observed in 15 of 39 patients (38%), whereas the remaining 24 patients (62%) exhibited at least one atypical feature for ADEM (Fig. 2). Among these 24 cases, 10 (42%) had one atypical feature, nine (38%) had two atypical features, and five (21%) presented with three atypical features. The most frequently observed atypical finding was the predominance of subcortical white matter lesions (17 of 39 patients, 44%), followed by the presence of small lesions (12 of 39 patients, 31%). Ten patients (26%) had both small and predominantly subcortical white matter lesions. These findings were compared with those reported by Baumann et al. [24], as summarized in Table 3.

**Table 2:** Summary of regions involved and widespread scores on magnetic resonance imaging

MRI regions involved	MOG-associated ADEM (*n* = 39)
Supratentorial white matter	39 (100%)
Thalamus/basal ganglia	26 (67%)
Brainstem	18 (46%)
Cerebellar	13 (33%)
Spinal	10/22 (45%)
LETM	9/10 (90%)
Centrally located^a^	9/10 (90%)
Widespread score (median/range)	2 (1–5)

*ADEM* acute disseminated encephalomyelitis, *LETM* longitudinally extensive transverse myelitis, *MOG* myelin oligodendrocyte glycoprotein, *MRI* magnetic resonance imaging

^a^Lesions involving the central gray matter on the axial image

**Table 3:** Summary of atypical magnetic resonance imaging features with comparison to the findings reported by Baumann et al. [24]

Atypical MRI features	This study (*n* = 39)	Study by Baumann et al. (*n* = 19)	*P*-value^a^
Small lesions	12 (31%)	0 (0%)	< 0.01
Subcortical white matter lesions^b^	17 (44%)	·	·
T1-hypointense lesions	4 (10%)	0 (0%)	0.29
Well-defined borders	2 (5%)	0 (0%)	1.00
Perpendicular^c^	2 (5%)	0 (0%)	1.00
Cortical lesions	5 (13%)	1 (5%)	0.65
Unilateral lesions	0 (0%)	0 (0%)	1.00
ADEM with ≥ 1 atypical MRI feature	24 (62%)	1 (5%)	< 0.01

*ADEM* acute disseminated encephalomyelitis, *MRI* magnetic resonance imaging

^a^Fisher’s exact test

^b^Lesions predominantly involving the subcortical white matter

^c^Lesions perpendicular to the ventricles

**Fig. 4:**
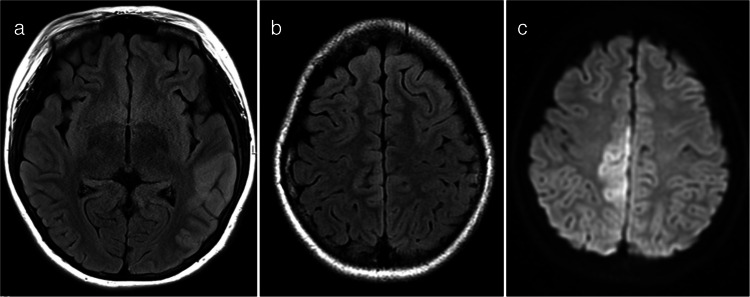
Magnetic resonance images illustrate myelin oligodendrocyte glycoprotein antibody-associated diseases with a cortical encephalitis pattern. **a** Axial fluid-attenuated inversion recovery image from an 11-year-old girl with seizures and drowsiness shows gyral swelling in the left temporal lobe (*arrows*), with no associated white matter lesion. **b**, **c** Axial fluid-attenuated inversion recovery (**b**) and diffusion-weighted (**c**) images from another 11-year-old girl. There is subtle hyperintensity in the right medial parietal cortex (*arrow* in **b**), with a diffusion-restrictive lesion in the same region (*arrow* in **c**). The lesion was initially misinterpreted as cortical infarction

**Fig. 5:**
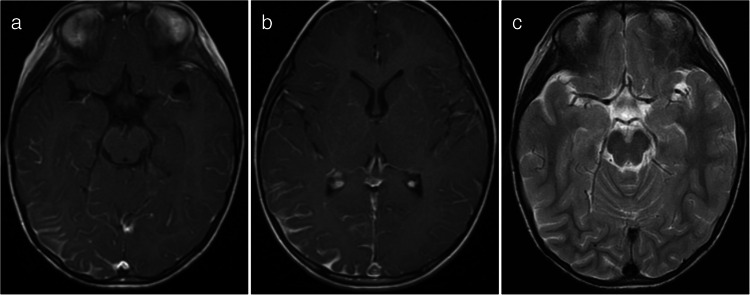
Magnetic resonance images illustrate a myelin oligodendrocyte glycoprotein antibody-associated disease with an aseptic meningitis pattern in a 3-year-old girl with seizure. **a**,** b** Post-contrast axial T1-weighted images obtained at the midbrain level (**a**) and at the foramina of Monro level (**b**) reveal localized leptomeningeal enhancement in the right occipito-temporal region. **c** Axial T2-weighted image at the same level as (**a**) shows no significant abnormalities in the brain parenchyma

**Fig. 6:**
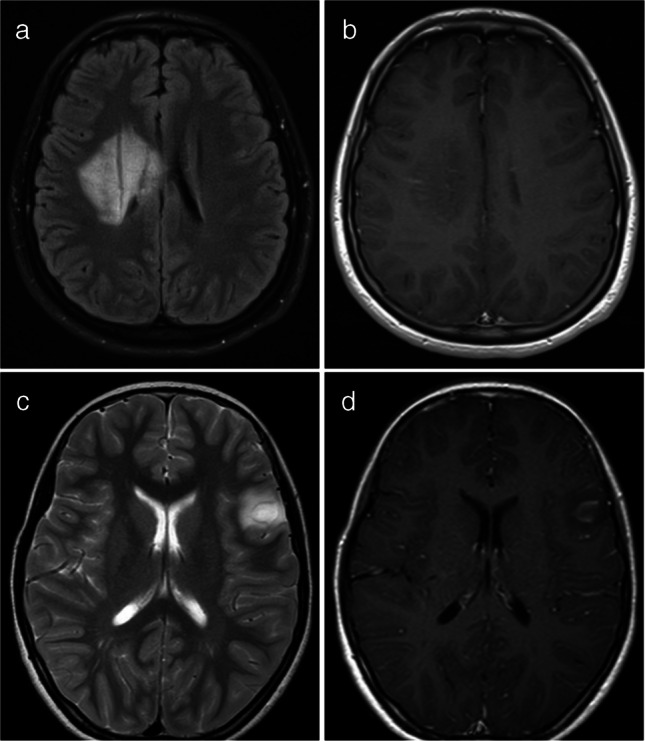
Magnetic resonance images illustrate myelin oligodendrocyte glycoprotein antibody-associated diseases with a tumefactive demyelination pattern. **a**, **b** Axial fluid-attenuated inversion recovery (**a**) and post-contrast T1-weighted (**b**) images from a 16-year-old girl show a large, focal hyperintense lesion in the right periventricular white matter with subtle linear enhancement. No notable mass effect is present. c, d Axial T2 (c) and T1 post-contrast (d) images from a 10-year-old girl with seizure demonstrate a focal cortical/subcortical lesion in the left frontal lobe. The lesion displays curvilinear, incomplete ring enhancement (arrow in d). This lesion was initially misdiagnosed as a small cortical abscess

**Fig. 7:**
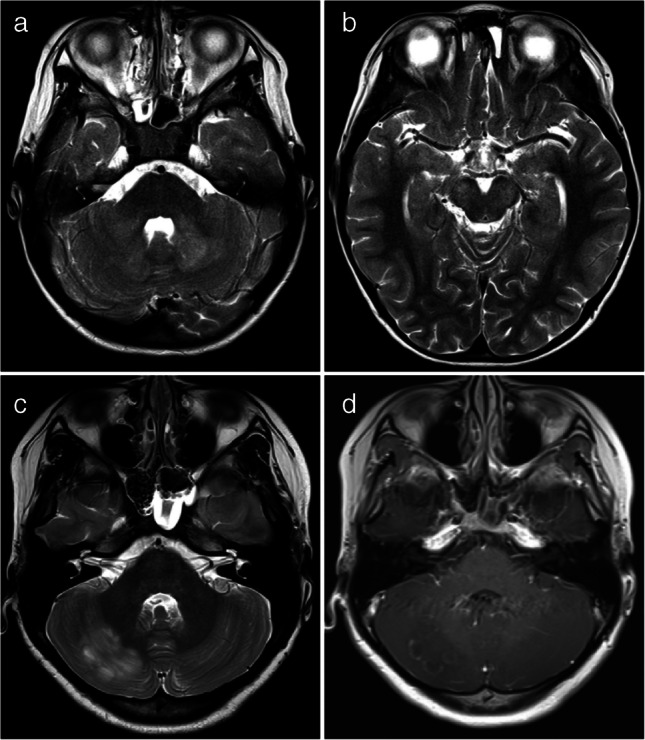
Magnetic resonance images illustrate myelin oligodendrocyte glycoprotein antibody-associated diseases with a cerebellitis/brainstem encephalitis pattern. **a**, **b** Axial T2-weighted images obtained at the fourth ventricle level (**a**) and the midbrain level (**b**) in a 5-year-old boy with dizziness show bilateral lesions in the cerebellar white matter (*arrows* in **a**) and midbrain (*arrows* in **b**). **c**,** d** Axial T2-weighted (**c**) and post-contrast T1-weighted (**d**) images in a 12-year-old girl with isolated cerebellitis show an ill-defined hyperintense lesion (*arrow* in **c**) in the right cerebellar cortex with curvilinear enhancement (*arrow* in **d**)

**Fig. 8:**
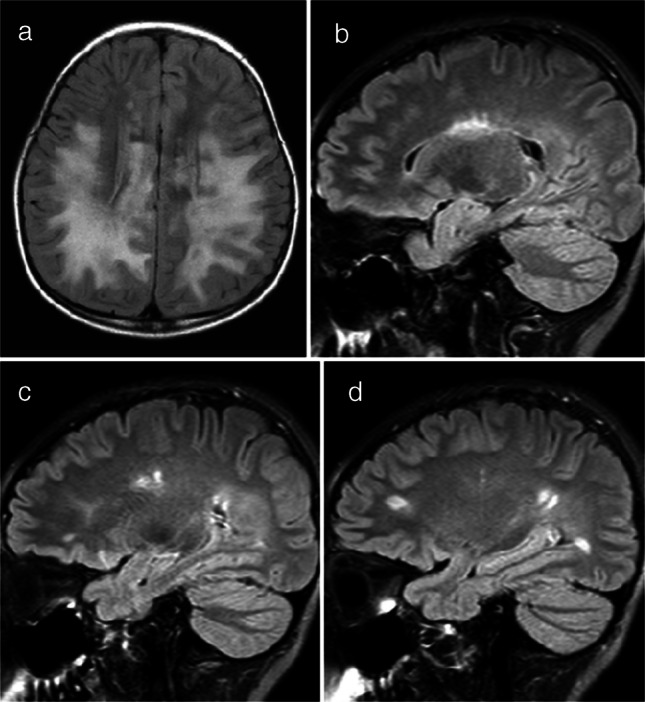
Magnetic resonance images illustrate myelin oligodendrocyte glycoprotein antibody-associated diseases of minor imaging patterns: a leukodystrophy-like pattern and a multiple sclerosis-like pattern. **a** Leukodystrophy-like pattern. Axial fluid-attenuated inversion recovery image from a 3-year-old girl with slurred speech, drooling, and right-sided weakness shows extensive, confluent, nearly symmetric white matter lesions resembling leukodystrophy. **b**,** c**,** d** Multiple sclerosis-like pattern. Three consecutive sagittal fluid-attenuated inversion recovery images from a 14-year-old boy demonstrate well-defined white matter lesions oriented perpendicular to the ventricles

A cortical encephalitis pattern was identified in three patients, and an aseptic meningitis pattern was seen in six patients (Figs. 4 and 5, respectively). Tumefactive demyelinating lesions were observed in three cases. These lesions were large (> 2 cm) and unifocal, resembling either neoplastic processes or localized abscesses (Fig. 6). The lesions were located in the periventricular white matter, thalamus, and subcortical white matter/cortex, respectively. Unlike typical tumors, these lesions did not demonstrate mass effect. A cerebellitis/brainstem encephalitis pattern was found in two patients (Fig. 7). One patient exhibited non-enhancing lesions in the cerebellar white matter and midbrain, while the other had poorly defined cerebellar cortical lesions with curvilinear enhancement. Among the miscellaneous patterns, one patient showed a leukodystrophy-like presentation (Fig. 8a), and another exhibited an MS-like pattern (Fig. 8b–d).

There was a statistically significant difference in age distribution between the ADEM group and non-ADEM groups (*P* < 0.01). Patients in the non-ADEM group were significantly older, with a median age of 11.5 years (range 3.3–17.8), compared to a median age of 5.2 years (range 1.3–11.5) in the ADEM group. Logistic regression analysis yielded an odds ratio of 0.65 (95% confidence interval (CI) 0.52–0.82), suggesting that each additional year of age was associated with a 35% decrease in the likelihood of presenting with ADEM. The optimal age cutoff to distinguish between the ADEM and non-ADEM groups was determined to be 7.6 years.

## Discussion

In children, MOGAD commonly presents as isolated or recurrent optic neuritis, isolated or combined myelitis, or ADEM. Since then, various atypical forms of brain involvement have been identified, and it is now recognized that, in contrast to optic neuritis or myelitis, brain involvement demonstrates a heterogeneous pattern that may include cortical encephalitis, aseptic meningitis, cerebellitis, and brainstem encephalitis. This study analyzed the radiologic features of anti-MOGAD presenting with cerebral syndromes involving the brain and brainstem in pediatric patients from a single center. While earlier studies have intermittently described imaging findings of MOGAD in presentations such as ADEM, cortical encephalitis, and others [4, 6, 17, 18, 25], we consider the present study to be valuable in offering a comprehensive review of neuroradiological features of MOGAD in children and in providing a broader radiologic overview of the disease.

In this study, ADEM was identified as the most frequent radiologic presentation of MOGAD with cerebral syndrome, accounting for 71% (39 out of 55) of the cases. Previous reports have also indicated that ADEM constitutes the largest subgroup (40–50%) in pediatric MOGAD and represents 70–80% of cases with cerebral involvement [4, 9, 26]. Characteristic MRI features of ADEM include diffuse, poorly defined, large (> 1–2 cm) lesions predominantly affecting the cerebral white matter [23]. Baumann et al. compared MOG-positive and MOG-negative ADEM and found that MOG-positive ADEM exhibited a more consistent pattern of typical ADEM, marked by large, bilateral, and widespread lesions. In contrast, MOG-positive ADEM in our cohort exhibited a higher frequency of atypical features, with small lesions being more prevalent (*P* = 0.005) compared to Baumann’s findings. Regarding lesion distribution, white matter involvement was primarily located in the subcortical regions in 44% of our MOG-ADEM cases. This pattern of preferential subcortical over periventricular or deep white matter involvement was also observed by Konuskan et al. in their MOG-ADEM cohort [27]. Consequently, in our study, approximately two-thirds of the cases (24 of 39, 62%) presented with atypical ADEM patterns, and about one-quarter (10 cases, 26%) displayed small, predominantly subcortical white matter lesions. Similarly, Dong et al. reported that at least one atypical ADEM feature was observed in 78.8% of their MOG-positive ADEM patients [28]. Therefore, we suggest that a large multicenter study is necessary to determine the true prevalence of typical versus atypical ADEM patterns in pediatric MOGAD. Nevertheless, a potential selection bias exists in our cohort. Patients with typical ADEM, who tend to recover quickly, may have been less likely to be referred to our institution, which is a national referral center. As a result, patients with more severe or atypical presentations were more likely to be included, possibly contributing to the relatively low proportion of “typical” ADEM in our study.

Although ADEM has traditionally been considered the predominant phenotype of MOGAD [4, 6], approximately 20% of pediatric MOGAD cases have been reported to present with a non-ADEM encephalitic phenotype [4]. In our study, 29% (16 out of 55) of MOGAD cases with cerebral syndromes did not meet the criteria for ADEM. Among these, aseptic meningitis was the second most common radiologic pattern following the ADEM phenotype, observed in six of the 55 patients (11%). This pattern is characterized on imaging by localized leptomeningeal enhancement or sulcal hyperintensity on FLAIR sequences, without associated brain parenchymal abnormalities. Consequently, such findings may be missed on pre-contrast MRI if post-contrast sequences are not performed. We suspect that some previously reported MOGAD cases with normal MRI findings may have actually represented aseptic meningitis [4, 9]. The relatively high frequency of aseptic meningitis in our cohort is concerning, as this subtype may not be identified under the 2023 MOGAD diagnostic criteria [14]. Based on our findings, we propose that aseptic meningitis should be recognized as a principal cerebral syndrome associated with MOGAD. Furthermore, our results support the routine inclusion of contrast-enhanced sequences in MRI protocol for the evaluation of suspected MOGAD.

Cerebral cortical encephalitis is another well-established non-ADEM phenotype of MOGAD [15]. Initially described in the seminal work by Ogawa et al., it is now recognized as one of the main non-ADEM cerebral syndromes [18]. Budhram et al. conducted a literature review of patients with serum MOG antibodies, encephalitis, and seizures and reported that MRI in these cases frequently showed unilateral cortical involvement. They introduced the acronym FLAMES (unilateral FLAIR-hyperintense lesions in anti-MOG-associated encephalitis with seizures) to describe this presentation [29]. Similarly, Wegener-Panzer et al. described 10 pediatric cases of encephalitis associated with MOG antibodies, in which MRI abnormalities were mainly confined to the cortex and deep gray matter, without cerebral white matter involvement [7]. This pattern was also identified in a small subset of our patients (three cases, 5%). Therefore, MOG-related encephalitis should be included in the differential diagnosis of cortical encephalitis. Recognition of this condition is essential, as it can easily be mistaken for viral encephalitis [16]. Another imaging pattern that may lead to diagnostic confusion is the tumefactive demyelination type, observed in three cases (5%). It typically presents as a solitary T2-hyperintense lesion resembling a tumor or abscess. Minimal or absent mass effect and evolution on follow-up MRIs may help distinguish it from neoplastic or infectious processes. Additionally, cerebellitis associated with MOGAD has been documented [30]. According to the 2023 MOGAD diagnostic criteria, such cases fall under the core clinical category of brainstem or cerebellar symptoms. In our study, one patient had lesions in both cerebellar white matter and the brainstem, while another presented with isolated cortical cerebellitis.

A leukodystrophy-like pattern, considered a rare presentation, was identified in one case in our study. This phenotype has been recently reported in pediatric MOGAD and appears to occur more frequently in younger children [21]. In line with this, our case involved a 3-year-old girl. Another distinct pattern observed in our study was a multiple sclerosis-like presentation, seen in just one patient. This was characterized by small, well-defined white matter lesions oriented perpendicular to the ventricles. MOG antibodies are rarely linked to multiple sclerosis in the pediatric population [4], which may explain why only one such case was identified in our cohort. In the study by Baumann M et al., multiple sclerosis-like patterns were absent in the MOG-positive ADEM group, while two cases (14%) were observed in the MOG-negative ADEM group [24]. The patient with the multiple sclerosis-like pattern in our study was lost to follow-up, so we could not determine whether this represented true multiple sclerosis. It is also noteworthy that no cases of limbic encephalitis were detected in our cohort. Although MOGAD can present as limbic encephalitis [31], bilateral medial temporal lobe involvement is considered atypical for MOGAD [25]. Furthermore, limbic encephalitis generally presents at an older age compared to other MOGAD subtypes such as ADEM and cortical encephalitis [31].

Finally, our study highlights a significant relationship between patient age and the radiological presentation of MOGAD with cerebral involvement. Younger children are more likely to exhibit the ADEM pattern, while older patients tend to present with atypical manifestations in children older than 7 years. Imaging remains critical for differentiating pediatric demyelinating diseases, which, besides MOGAD, include pediatric multiple sclerosis, aquaporin-4-antibody-positive NMOSD, and other less clearly defined categories. Radiologists must also consider a broad differential diagnosis when evaluating suspected MOGAD, including infectious encephalitis, CNS vasculitis, CNS hemophagocytic lymphohistiocytosis, brain tumors, and neurometabolic disorders [32, 33].

This study has several significant limitations. First, we did not include a comparison group of children who met the diagnostic criteria but were negative for MOG antibodies. However, the objective of this study was to characterize the diverse radiologic features of MOGAD, rather than to compare findings with MOG-negative cases. Second, follow-up imaging was not analyzed. In our cohort, some patients experienced relapses with different radiologic patterns. Since incorporating follow-up data would add complexity, we consider this a topic better suited for future research. Third, optic neuritis was not evaluated in the study. Although optic neuritis is a common manifestation of pediatric MOGAD and may co-occur with cerebral syndromes, it was not evaluated here due to the lack of dedicated imaging sequences for the optic nerves in most cases. Fourth, given the retrospective design of this study, certain clinical data, such as electroencephalography findings, were incomplete or unavailable. Fifth, the possibility of selection bias must be acknowledged. As a major national tertiary center, our institution is more likely to have received patients with severe symptoms, while patients with milder clinical and radiologic presentations may have been underrepresented. Therefore, we do not believe this study captures the full radiologic spectrum of pediatric MOGAD, and a large-scale multicenter study is warranted.

## Conclusion

In summary, pediatric MOGAD involving the brain presents with a variety of imaging patterns. While the ADEM phenotype was most frequently observed, it commonly displayed atypical features, particularly small, subcortical white matter lesions. We also described imaging characteristics of less commonly reported phenotypes, including cerebral cortical encephalitis, aseptic meningitis, tumefactive demyelinating lesions, and cerebellitis. Together, these variants constitute a considerable portion of brain-involved MOGAD cases and occur more frequently in older children. Recognizing the broad imaging spectrum of MOG-associated brain disease is essential for achieving an accurate diagnosis.

## Supplementary Information

Below is the link to the electronic supplementary material.

Supplementary file1 (DOCX 23 KB)

## Data Availability

The datasets generated during and/or analyzed during the current study are available from the corresponding author on reasonable request.
