# Supplementary material for: Neuroimaging spectrum of myelin oligodendrocyte glycoprotein antibody-associated disease with brain involvement: description of various cerebral syndromes
Source: Pediatr Radiol. 2025 Aug 11;55(10):2115–26. doi: 10.1007/s00247-025-06342-y (PMC12513890; doi:10.1007/s00247-025-06342-y)
Supplement: Supplementary file 1 — Supplementary file1 (DOCX 23 KB) [file 247_2025_6342_MOESM1_ESM.docx]

Supplementary table Clinical symptoms according to the subtypes

|  | ADEM  (*n*=39) | Cortical encephalitis (*n*=3) | Aseptic meningitis  (*n*=6) | Tumefactive demyelination (*n*=3) | Cerebellitis/brainstem encephalitis (*n*=2) | Leukodystrophy-like  (*n*=1) | MS-like  (*n*=1) |
| --- | --- | --- | --- | --- | --- | --- | --- |
| Fever | 24 (62%) | 1 (33%) | 6 (100%) | 3 (100%) | 2 (100%) | 0 (0%) | 0 (0%) |
| Headache | 8 (21%) | 0 (0%) | 4 (67%) | 3 (100%) | 0 (0%) | 0 (0%) | 1 (100%) |
| Seizure | 11 (28%) | 3 (100%) | 4 (67%) | 1 (33%) | 0 (0%) | 0 (0%) | 1 (100%) |
| Gait instability/ataxia | 27 (69%) | 2 (67%) | 2 (33%) | 0 (0%) | 2 (100%) | 1 (100%) | 0 (0%) |
| Psychiatric symptom | 12 (31%) | 0 (0%) | 1 (17%) | 1 (33%) | 1 (50%) | 0 (0%) | 0 (0%) |
| Dysphasia | 15 (38%) | 2 (67%) | 1 (17%) | 0 (0%) | 1 (50%) | 0 (0%) | 0 (0%) |
| Movement disorder | 13 (33%) | 1 (33%) | 2 (33%) | 1 (33%) | 0 (0%) | 1 (100%) | 0 (0%) |
| Brainstem dysfunction | 6 (15%) | 0 (0%) | 1 (17%) | 0 (0%) | 1 (50%) | 0 (0%) | 0 (0%) |
| Focal Weakness | 19 (49%) | 3 (100%) | 4 (67%) | 0 (0%) | 2 (100%) | 1 (100%) | 0 (0%) |
| Memory dysfunction | 1 (3%) | 0 (0%) | 0 (0%) | 1 (33%) | 0 (0%) | 0 (0%) | 0 (0%) |
| Altered consciousness | 16 (41%) | 1 (33%) | 4 (67%) | 2 (67%) | 0 (0%) | 0 (0%) | 1 (100%) |

Numbers (%), *ADEM* acute disseminated encephalomyelitis, *MS* multiple sclerosis
